# Preliminary In Vitro Studies on *Corynebacterium urealyticum* Pathogenetic Mechanisms, a Possible Candidate for Chronic Idiopathic Prostatitis?

**DOI:** 10.3390/microorganisms8040463

**Published:** 2020-03-25

**Authors:** Daria Nicolosi, Carlo Genovese, Marco Alfio Cutuli, Floriana D’Angeli, Laura Pietrangelo, Sergio Davinelli, Giulio Petronio Petronio, Roberto Di Marco

**Affiliations:** 1 Department of Biomedical and Biotechnological Sciences—Microbiology Section, Università degli Studi di Catania, 95100 Catania, Italy; dnicolosi@unict.it (D.N.); gnv.carlo@gmail.com (C.G.); fdangeli@unict.it (F.D.); 2 Department of Medicine and Health Sciences “Vincenzo Tiberio”, Università degli Studi del Molise—III Ed Polifunzionale, 86100 Campobasso, Italy; m.cutuli@studenti.unimol.it (M.A.C.); laura.pietrangelo@unimol.it (L.P.); sergio.davinelli@unimol.it (S.D.); roberto.dimarco@unimol.it (R.D.M.)

**Keywords:** *Corynebacyerium urealyticum*, prostate, urinary infections, LNCaP, adhesion, acetohydroxamic acid

## Abstract

*Corynebacterium urealyticum* is a well-known opportunistic uropathogen that can occur with cystitis, pyelonephritis, and urinary sepsis. Although a wide variety of coryneform bacteria have been found from the male genital tract of prostatitis patients, only one clinical case of prostatitis caused by *C. urealyticum* has been reported. The aim of this study was to evaluate the in vitro tropism of *C. urealyticum* towards LNCaP (lymph node carcinoma of the prostate) human cells line and the influence of acetohydroxamic acid as an irreversible urease inhibitor on different aspects of its pathogenicity by means of several in vitro tests, such as the determination and analysis of growth curves, MTT (3-(4,5-dimethylthiazol-2-yl)-2,5-diphenyltetrazolium bromide) assay, the production of biofilms, and adhesion to LNCaP and HeLa cell lines. Results have brought new pieces of evidence on the in vitro tropism of *C. urealyticum* for the human prostate cell line LNCaP and the therapeutic use of the irreversible urease inhibitors such as acetohydroxamic acid (AHA), not only as enzyme blockers to facilitate the removal of encrustations but also as modulators of some pathogenic mechanisms. These interesting preliminary data allow us to assert that there is a real possibility that *C. urealyticum* is a new candidate for chronic idiopathic prostatitis.

## 1. Introduction

*Corynebacterium urealyticum* (*C. urealyticum*), formerly known as coryneform CDC 79 group D2, belongs to the family *Corynebacteriaceae* and the genus *Corynebacterium* [1]. The first warning about these dangerous bacteria was made by Luis Cifuentes in 1947, but its identification and recognition at an international level are due to the subsequent efforts Dr. F. Soriano [2].

*C. urealyticum* is a problematic identification bacterium, and is a slow-growing bacillus with diphtheroid morphology, Gram-positive, aerobic and facultative anaerobic, generally non-motile, not saccharolytic, and lipophilic with a strong urease activity [3].

The peculiar conditions required for the isolation and cultivation in vitro of *C. urealyticum* often make it undetectable to routinely urine cultures. However, it can be isolated after 48 hours of incubation at 35–37 °C in 10% CO_2_ on blood agar plates, where it shows a precise morphology of the colonies [4].

This species has been considered as part of the physiological commensal human microbiota of the skin (isolated in about 12–30% of cases), upper respiratory tract mucosae, urinary tract, and conjunctival mucosae membrane. Nevertheless, it has been reported as an opportunistic nosocomial pathogen. The more frequent risk factors are older age, prolonged hospitalization, immunosuppression, invasive urological procedures, broad-spectrum antibiotic prophylaxis or therapy, and underlying genitourinary disorders that can cause urinary pathologies such as acute and chronic UTIs (urinary tract infections) [5].

Among chronic UTIs, the most frequent chronic infections caused by *C. urealyticum* is a peculiar form of cystitis named “encrusted cystitis”. This is a condition of chronic ulcerative bladder inflammation that was described for the first time in 1914 by J. Francois [6]. Due to the difficulties encountered in isolation and cultivation in vitro, the diagnosis of *C. urealyticum*-encrusted cystitis is often overlooked or delayed. For these reasons, this pathology is little known and the pathogenicity of *C. urealyticum* is often questioned as contamination [2,7].

Besides encrusted cystitis, *C. urealyticum* is the causative agent of another severe chronic UTI—encrusted pyelitis. As is the case with encrusted cystitis, this pathology is also characterized by the presence of obstructive deposits on the renal pelvis wall and often diagnosed only when the pelvis is open during a urological procedure [4].

The etiology of these chronic UTIs are correlated with the *C. urealyticum* ability to produce urease, an enzyme that, breaking down the urea and developing ammonium’s ions, alkalizes the urine with the formation of obstructive crystals (ammonium magnesium phosphate: NH_4_MgPO_4_) deposited on the bladder mucosae. Therefore, this pathologic bladder environment is considered the main cause of tissue damage [3].

Only recently has *C. urealyticum* also been associated with prostatitis. In 2018, Pardo Martínez et al. illustrated a clinical case of a patient with a history of prostatic neoplasia treated with radiotherapy that showed a significant calcification at the prostate level. An initial examination of the urine revealed an *E. coli* infection with an alkaline pH, with only the microbiological analysis of the pathological prostate tissue revealing infection by *C. urealyticum*. In addition, in this case, the authors pointed out the difficulty of a correct diagnosis for the *C. urealyticum* infection due to its slow growth and the challenge of identification and in vitro isolation [8].

The problem of identifying the causative agent in prostatitis is well known to both clinicians and microbiologists. Indeed, if the microbiological diagnosis of acute bacterial prostatitis is considered a laboratory practice, on the other hand, chronic idiopathic prostatitis is often a difficult challenge for the microbiology laboratory due to some pathogens such as the coryneform bacteria that require particular conditions and long incubation times to grow in vitro.

When correctly identified, along with the antibiotic therapy, the therapeutic approach for encrusted cystitis also provides the elimination of the plates by endoscopic resection and acidification of the urine. Therefore, antibiotic therapy is a part of the treatment but, especially in the case of altered general condition, the antibiotic treatment alone may be considered if the diagnosis is early [9].

Due to natural resistance (resistance to beta-lactamases, sensitivity preserved to glycopeptides) [10], the antibiotics usually prescribed are vancomycin and teicoplanin for 4 to 6 weeks [7,11].

*Corynebacterium urealyticum* is often a multi-drug-resistant organism. As most of the clinical strains are isolated in the nosocomial environment, we can reasonably assume that high antibiotic resistance can be acquired from the hospital environment [12]. As proof of this, during the treatment, development of resistance versus beta-lactam antibiotics, fluoroquinolones, macrolides, rifampin, tetracycline, and gentamicin has been observed, with a significant risk of failure in therapy and development of complications [13,14,15]. Although many drugs fail to penetrate the prostate tissue [16], vancomycin, despite its nephrotoxicity risk in elderly patients [17], it is one of the elective drugs for therapy against *C. urealyticum* infection [7,11] that manages to achieve equal or higher tissue concentrations in the prostate than those of serum [18].

Nevertheless, from as early as 1991, according to Marty et al. [19], it has been announced that many aspects of *C. urealyticum* pathogenicity still need to be revealed. The adhesively of this bacterium does not seem merely mediated by pili. However, *C. urealyticum* expresses a pilus proteinaceous with a certain structural similarity with the pilus of *C. diphtheria* [20]; there is evidence of some strains without pili or with low numbers of them that are still able to adhere to the urothelium surface [19]. Indeed, the intense urease activity plays a crucial synergistic role in its pathogenicity [21].

Furthermore, the urease activity of other uropathogenic bacteria, such as *Klebsiella* spp. and *Proteus mirabilis*, has been extensively studied and it has been demonstrated that it plays a relevant role for the pathogenic activity, providing urea as a nutrient, obstruction of the ureters, and epithelial cytotoxicity induced by ammonia. However, the stones produced by these pathogens do not cause encrustation on the mucosa within the urinary tract like *C. urealyticum* [22,23].

The therapeutic use of irreversible urease inhibitors such as acetohydroxamic acid (AHA) for the reduction of urinary stones by uropathogenic bacteria has been widely demonstrated [24,25]. Moreover, AHA is particularly useful in reducing the in vitro deposition of calcium and magnesium salts on silicone catheters exposed to urine with *P. mirabilis* infection [26].

Although AHA is frequently used in the treatment of *C. urealyticum* infection for its bacteriostatic effect and its synergic action with antibiotics [27], at present there are no scientific publications that have evaluated the effect of AHA on *C. urealyticum* pathogenicity by in vitro assays.

For all these reasons, the aim of this study was to evaluate the in vitro tropism of *C. urealyticum* towards LNCaP (lymph node carcinoma of the prostate) prostate cell line and the influence of two different concentrations of AHA (0.25 µg/mL and 0.50 µg/mL) as an irreversible urease inhibitor on different aspects of its pathogenicity and to determinate if dose–response activity could be relevant. To this end, several in vitro tests, in the presence of two concentrations of acetohydroxamic acid (AHA), were set up, including the determination and analysis of growth curves, MTT (3-(4,5-dimethylthiazol-2-yl)-2,5-diphenyltetrazolium bromide) assay, the production of biofilms, and adhesion to LNCaP and HeLa cell lines.

## 2. Materials and Methods

### 2.1. Chemicals

AHA and crystal violet (CV) were purchased from Merck (Darmstadt, Germany, EU), whereas MTT (3-(4,5-dimethylthiazol-2-yl)-2,5-diphenyltetrazolium bromide) and Tween 80 were from Thermo Fisher Scientific Inc. All chemicals were used without further purification. Deionized distilled water obtained by Millipore system was used for the preparation of solutions.

### 2.2. Bacterial Strains

*Corynebacterium urealyticum* ATCC 43042 (isolated from bladder stone), *Corynebacterium urealyticum* ATCC 43043 (isolated from urine), and *Corynebacterium urealyticum* ATCC 43044 (isolated from urine) were purchased from the American Type Culture Collection (Rockville, MD). Bacterial strains were cultured in Tryptic Soy Broth Medium (bioMérieux) with 0.1% Tween 80 (Thermo Fisher Scientific Inc.) aerobically with shaking at 35.5 °C.

### 2.3. Cell Cultures

The human adenocarcinoma HeLa cell line was purchased from the American Type Culture Collection (Rockville, MD). Cells were maintained in Eagle’s minimum essential medium (Merck-Darmstadt, Germany, EU) with 10% of fetal bovine serum (Gibco—Thermo Fisher Scientific, USA) and 1% penicillin/streptomycin (p/s) at 37 °C with 5% CO_2_ humidified incubator. Human prostatic cancer LNCaP cells were also from ATCC. Cells were cultured in Roswell Park Memorial Institute (RPMI)-1640 Medium (Gibco—Thermo Fisher Scientific, USA), supplemented with 10% fetal bovine serum (Gibco—Thermo Fisher Scientific, USA) and 1% penicillin/streptomycin (p/s) in 95% humidified air, with 5% CO_2_ at 37 °C. Cells were passaged once a week after trypsinization and replaced with new medium twice weekly.

### 2.4. Influence of AHA on In Vitro Growth Curves by Cell Counting

The influence of AHA on in vitro growth curves was carried out according to the method proposed by Fuochi et al. [28] with some changes. Culture medium composition, temperature, and incubation time were adapted for optimal in vitro growth of *C. urealyticum*.

A bacterial suspension of 0.5 McFarland, equivalent to at 1.5 × 10^8^ Colony-Forming Unit (CFU)/mL, for three different *C. urealyticum* strains (*C. urealyticum* ATCC 43042; *C. urealyticum* ATCC 43043; *C. urealyticum* ATCC 43044) was made after 72 h subculture in Trypticase soy broth (TSB) (bioMérieux) enriched with 0.1% Tween 80 (Thermofisher Scientific Oxoid), measuring OD at 600 nm (Model 680 Microplate Reader, Bio-Rad, Milan, Italy). A series of dilutions were prepared in order to obtain a final concentration of 8 × 10^7^ CFU/mL in TSB 0.1% Tween 80. AHA was added in four different concentrations (0.25 µg/mL and 0.50 µg/mL). Inoculated bottles were incubated aerobically with shaking at 35.5 °C; 100 µL of each dilution was taken every 60 min for 72 h for cell counting. For this purpose, serial 10-fold dilutions in sterile saline (NaCl 0.85% w/v) were performed, and each dilution was spread on TSB 0.1% Tween 80. All measurements were performed in triplicate in six independent experiments.

### 2.5. In Vitro Bacterial Grown Curve Analysis

The analysis of the bacterial growth curves was performed according to the method proposed by Fuochi et al. [28] with some changes. A new parameter (∆X_CFU50_) was proposed and a new mathematical function (sigmoidal dose–response) was used for data analysis.

The percentage of inhibition of bacterial growth for each strain was calculated according to the following formula:

(1) $$\%\;\mathrm{of}\;\mathrm{inhibition}=100-\frac{\mathrm{Strain}\;Y_{top}\;\mathrm{at}\;0.25\;\mathrm{or}\;0.50\;\mathrm{mg}/\mathrm{mL}\;\mathrm{AHA}}{\mathrm{Strain}\;Y_{top}\;\mathrm{Control}\times 100}$$

where *Y_top_* is the maximum value of CFU/mL obtained for each bacterial strain tested (alone or treated with the two concentration of AHA).

The time delay (expressed in hours) in reaching the exponential growth phase for each strain treated with the two AHA concentrations tested compared to the untreated strain (∆X_50CFU_) was calculated according to the following formula:

∆X_CFU50_ = (Strain ∆X_CFU50_ at 0.25 or 0.50 mg/mL AHA) − (Strain Strain ∆X_CFU50_ Control) (2)

where X_CFU50_ is the X value (time in hours) when the CFU/mL count is halfway between the bottom and top of the curve, or halfway through the exponential growth phase.

All parameters were calculated by analyzing in vitro the bacterial growth curves of all strains tested with sigmoidal dose–response (variable slope) function by Prism GraphPad Software 6 (GraphPad Software, California, CA, USA) (Table 1).

### 2.6. MTT Assay

By 3-[4, 5-dimethylthiazol-2-yl]-2, 5-diphenyl tetrasodium bromide (MTT) assay, we evaluated LNCaP cell viability, grown with or without different concentrations of AHA (0.25 and 0.50 mg/mL) and in presence or absence of three different *C. urealyticum* strains: *C. urealyticum* ATCC 43042, *C. urealyticum* ATCC 43043, and *C. urealyticum* ATCC 43044, for 24 hours, as reported by Motta et al. (2015). Briefly, LNCaP cells were seeded in 96-well plates at a density of 1.5 × 10^4^ per well and incubated overnight at 37 °C before experiments. Successively, cells were infected with 1.5 × 10^3^ CFU/mL of each *C. urealyticum* bacterial suspension and, simultaneously, treated with AHA at the concentrations mentioned above for 24 h. After the incubation period, 10μl of MTT reagent (5 mg/mL) was added to each well and the cells were incubated for 3 h at 37 °C. The formazan crystals were solubilized with 100 μL Dimethyl sulfoxide DMSO and plates were shaken for 10 min. The absorbance was measured at 570 nm with a plate reader (Synergy 2-bioTek). The tests were performed in triplicate in three independent experiments.

### 2.7. In Vitro Biofilm Formation Assay

Biofilm formation assay was performed according to modified Soriano and Gomes methods [13,29]. A total of 200 μL of bacterial suspension for each strain (*C. urealyticum* ATCC 43042, *C. urealyticum* ATCC 43043, *C. urealyticum* ATCC 43044) containing 10^6^ CFU/mL in Trypticase soy broth (TSB) (bioMérieux) enriched with 0.1% Tween 80 (Thermofisher Scientific Oxoid) was inoculated in wells and incubated at 35.5 °C for 72 h. After the incubation, the wells were discharged and washed twice with 200 μL of phosphate-buffered saline (PBS). Then, 200 μL per well of 1% crystal violet (CV) (Merck) solution was added for 15 minutes. The wells were discharged and washed twice with 200 μL of PBS. After removing the washing solution, the microplates were air dried and 200 μL of glacial acetic acid was added per well to dissolve the biofilm-bound CV. Absorbance was measured at 570 nm. Each experiment was performed with two different concentrations of AHA (0.25 and 0.50 mg/mL) to evaluate its effect on in vitro biofilm formation. Cut-off optical density (ODc) represented the OD mean of blank wells (negative control). On the basis of ODc, the strains were classified as non-adherent (OD ≤ ODc), weakly adherent (ODc < OD ≤ 2 × ODc), moderately adherent (2 × ODc < OD ≤ 4 × ODc), and strongly adherent (4 × ODc ≤ OD). The tests were performed in triplicate in six independent experiments.

### 2.8. Effect of AHA on C. urealyticum Adhesion to Human LNCaP and HeLa Cell Lines

Bacterial adherence on human adenocarcinoma (HeLa) and on human prostatic cancer (LNCaP) cell lines was determined according to the method proposed by Gomes et al. [29] with slight changes. Culture media and incubation conditions were adapted for the in vitro growth of both *C. urealyticum* and LNCaP and HeLa cell lines. Cells were grown in full medium before infection. A total of 100 μl of bacterial suspension for each strain (*C. urealyticum* ATCC 43042, *C. urealyticum* ATCC 43043, *C. urealyticum* ATCC 43044) were washed twice with phosphate-buffered saline (PBS) (Gibco) and resuspended in both Minimal Essential Medium (MEM) and RPMI medium, without p/s, to a final concentration of 3 × 10^3^ CFU/mL. Cell monolayers were grown to approximately 95% confluence in 24-cell tissue culture plates (Corning), washed three times with PBS, and were infected with 1 mL per well of bacterial suspension and simultaneously treated with different concentrations of AHA (0.25 and 0.50 mg/mL). After incubation in 5% CO_2_ at 37 °C for 2 h, infected monolayers were washed three times with PBS. Afterwards, the cells were scraped off in 1 mL of PBS and the total bacterial count was determined by the plate count method. Dilutions of the suspensions in the 1:10 ratio were made and plated on brain heart infusion agar (BHI) with 1% Tween 80 to enumerate cell-attached bacteria. Each experiment was performed with two different concentrations of AHA (0, 25, and 50 mg/mL) to evaluate its effect on cell adhesion. Results are expressed as mean ± standard deviation of triplicate in six independent experiments in triplicate.

### 2.9. Statistical Analysis

Data are expressed as mean ± standard deviation (SD) for three replicates of three independent experiments (i.e., biological and technical triplicates). Statistical significance was analyzed by two-way ANOVA test. The goodness of fit for sigmoidal dose–response (variable slope) functions was verified by *R*^2^ values >0.99 for all the data tested (Table 1).

## 3. Results

### 3.1. Influence of AHA on in vitro Growth Curves by Cell Counting and Analysis

**Figure 1:**
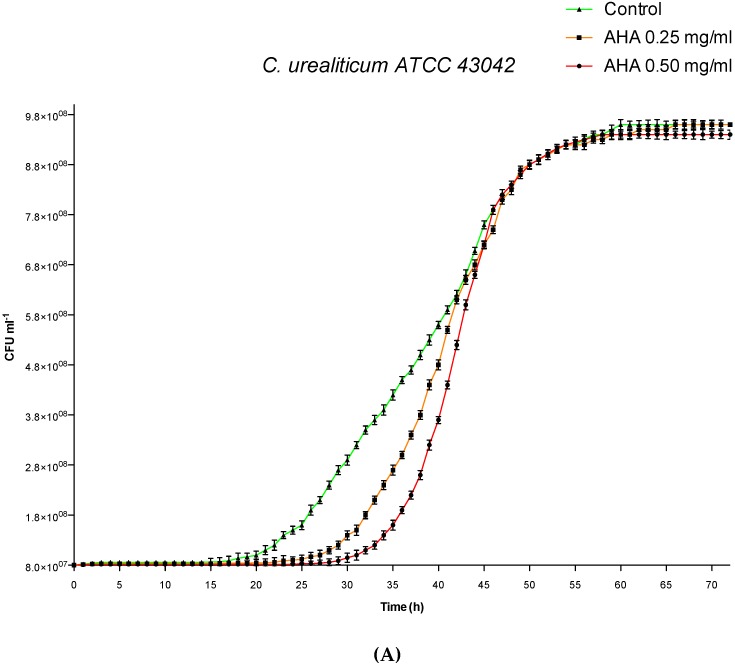

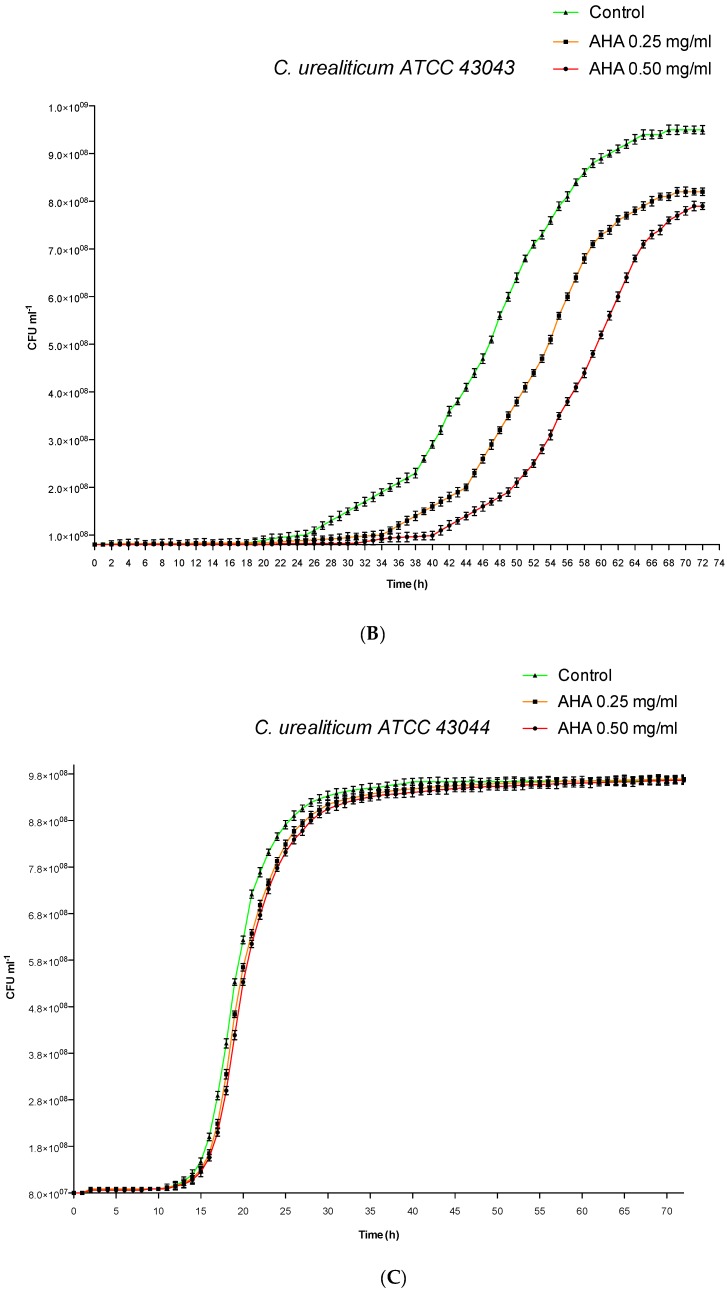
Influence of AHA on in vitro growth curves by cell counting. Green lines: untreated controls. Orange lines: strains treated with AHA 0.25 mg/mL. Red lines: strains treated with AHA 0.50 mg/mL. (**A**) *C. urealyticum* ATCC 43042. (**B**) *C. urealyticum* ATCC 43043. (**C**) *C. urealyticum* 43044. The bars represent means ± SD of independent experiments performed in triplicate (SD = standard deviation).

As illustrated in Figure 1, for the three tested strains of *C. urealyticum*, the impact of AHA on the in vitro growth curves was concentration- and strain-dependent. Concerning *C. urealyticum* ATCC 43044, the two tested concentrations of AHA (0.50 and 0.25 mg/mL) exerted a minimal impact on the in vitro growth curves (Figure 1C). In contrast, the administration of AHA in *C. urealyticum* ATCC 43042 and 43043 showed a delay in reaching the growth exponential phase proportional to the concentrations of AHA compared to the untreated control (Figure 1A,B). Inhibition rates (% of inhibition) and delay times in the exponential phase (∆X_CFU50_) of the in vitro growth for the three strains of *C. urealyticum* tested are shown in Table 1.

### 3.2. Effects of AHA Treatments and C. urealyticum Infections on LNCaP and HeLa Cell Viability

**Figure 2:**
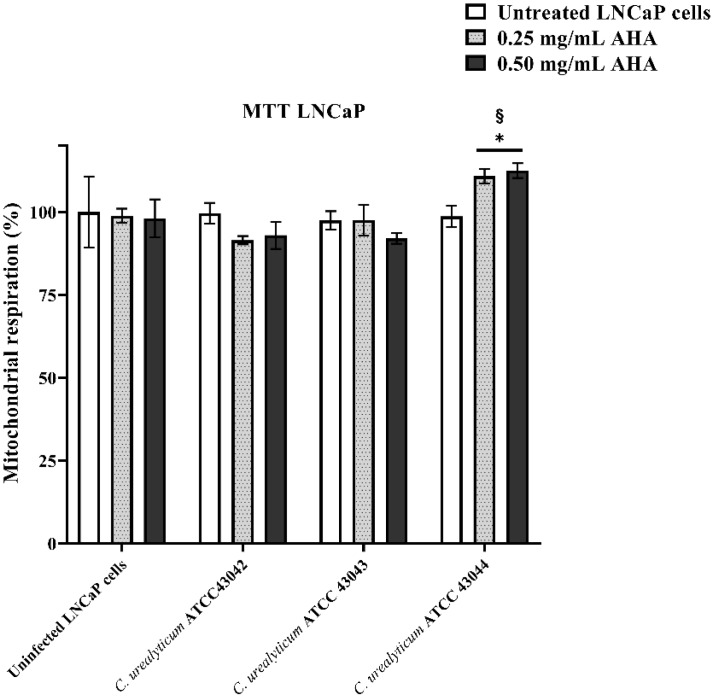
Effects of *C. urealyticum* infections and AHA treatments on LNCaP (lymph node carcinoma of the prostate) cell viability. Histograms show mitochondrial respiration rate of LNCaP cells grown in absence (uninfected LNCaP cells) or presence of *C. urealyticum* strains (ATCC 43042; ATCC 43043; ATCC 43044), without (white histograms) or with two different concentrations (0.25 µg/mL light grey histograms with dots and 0.50 µg/mL black histograms) of AHA for 24h. Results are expressed as percentage. The bars represent means ± SD of three independent experiments performed in triplicate (SD = standard deviation). Statistically significant differences, determined by two-way analysis of variance ANOVA, are indicated: * *p* < 0.05 versus untreated; § *p* < 0.05 versus uninfected.

In order to evaluate the effects of *C. urealyticum* infections and AHA treatments on LNCaP mitochondrial respiration and before testing the efficacy of AHA in reducing biofilm formation and *C. urealyticum* adhesion, we performed MTT assay. Therefore, LNCaP cells, grown alone or in the presence of the three *C. urealyticum* strains (*C. urealyticum* ATCC 43042; *C. urealyticum* ATCC 43043; *C. urealyticum* ATCC 43044) were simultaneously treated with 0.25 mg/mL or 0.50 mg/mL of AHA for 24 h. At this time point, no difference in LNCaP mitochondrial respiration rate was observed when the uninfected cells were treated with AHA at the concentrations mentioned previously (Figure 2). Analogously, the infection of cells with all tested *C. urealyticum* strains did not affect the mitochondrial respiration rate of untreated LNCaP. Furthermore, also the concomitant infection and AHA treatment did not determine any change in LNCaP cell viability except for *C. urealyticum* ATCC 43044. Indeed, in this case, the treatment with AHA, at the two tested concentrations, was able to enhance mitochondrial respiration rate of LNCaP cells infected with *C. urealyticum* ATCC 43044 compared to both untreated and uninfected cells. No variation in mitochondrial respiration rate was observed in HeLa cells for all experimental conditions.

### 3.3. Effects of AHA Treatments on in Vitro Biofilm Formation Assay

**Figure 3:**
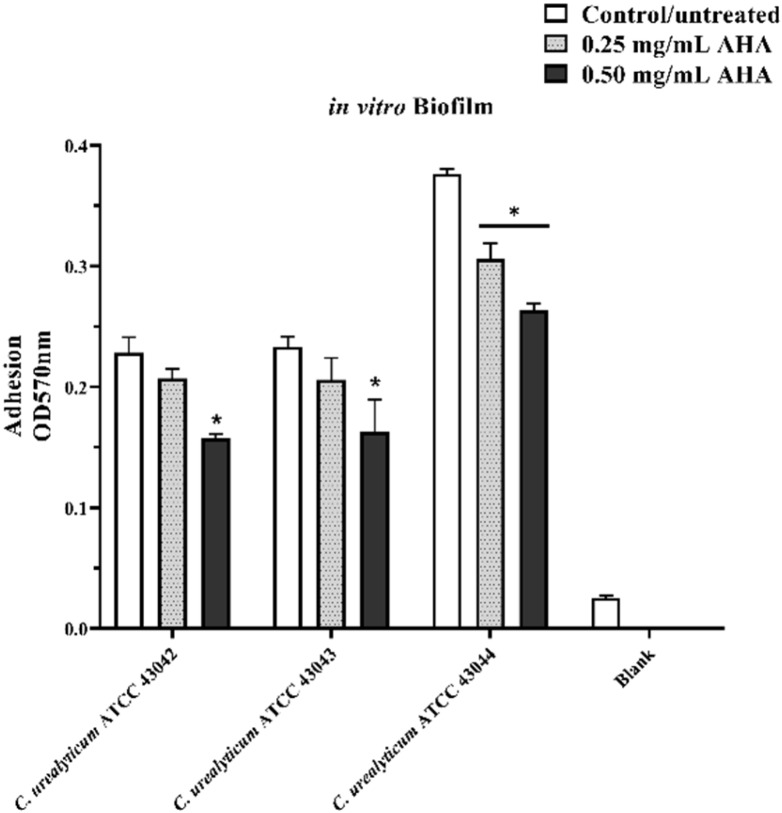
Effects of AHA treatments on in vitro biofilm formation assay. In vitro biofilm formation from the three strains of *C. urealyticum* tested determined by crystal violet (CV) staining. Blank: negative control without bacterial strains. White histograms: strains not treated with AHA. Light grey histograms with dots: strains treated with 0.25 mg/mL AHA. Black histograms: strains treated with 0.50 mg/mL AHA. The bars represent means ± SD of three independent experiments performed in triplicate (SD = standard deviation). Statistically significant differences, determined by two-way analysis of variance ANOVA, are indicated: * *p* < 0.05 versus control/untreated.

The results of in vitro biofilm formation assay for the three strains of *C. urealyticum* tested (Figure 3, white histograms) agree with those reported by Soriano et al. [13]. In the absence of AHA, the OD570 biofilm/OD570 control ratio was >4 for all tested strains (strongly adherent). Administration of AHA was able to reduce the in vitro biofilm formation of all tested bacteria in a concentration-dependent manner. For *C. urealyticum* ATCC 43042 and *C. urealyticum* ATCC 43043, the addition of 0.50 mg/mL of AHA determined a significant reduction of biofilm formation compared to the control condition (untreated bacteria). Concerning *C. urealyticum* ATCC 43044, the treatment with AHA significantly reduced the in vitro biofilm formation at both concentrations. Although there was a reduction in biofilm formation, both AHA concentrations tested (0.25 and 0.50) failed to significantly reduce the OD570 biofilm/blank (negative control) ratio, which remained above 4 for all three strains tested.

### 3.4. Effect of AHA on C. urealyticum Adhesion to Human LNCaP and HeLa Cell Lines

**Figure 4:**
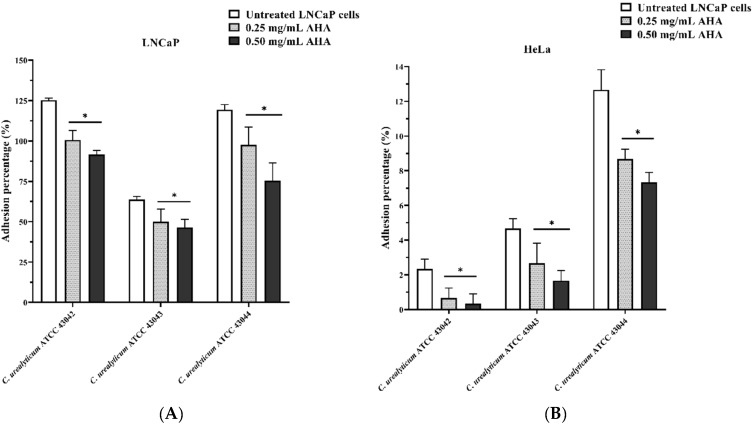
Effect of AHA on *C. urealyticum* adhesion to human LNCaP (**A**) and HeLa (**B**) cell lines. White histograms: strains not treated with AHA. Light grey histograms with dots: strains treated with 0.25mg/mL AHA. Black histograms: strains treated with 0.50 mg/mL AHA. Results are expressed as percentage. The bars represent means ± SD of three independent experiments performed in triplicate (SD = standard deviation). Statistically significant differences, determined by two-way analysis of variance ANOVA, are indicated: * *p* < 0.05 versus control/untreated.

Figure 4 shows the results of the adhesion on the two different cell lines (LNCaP: Figure 4A, and HeLa: Figure 4B) for the three strains of *C. urealyticum* tested. All strains not treated with AHA (white histograms) demonstrated higher tropism towards LNCaP cells (Figure 4A) with adhesion values of around 125% for *C. urealyticum* ATCC 43042, 64% for *C. urealyticum* ATCC 43043, and 119% for *C. urealyticum* ATCC 43044. On the other hand, adhesion on HeLa cells was much lower with a maximum value of 13% for *C. urealyticum* ATCC 43044. Treatment with AHA significantly reduced adhesion in a concentration-dependent manner for all strains tested on both cell lines (Figure 4A,B, light grey with dots and black histograms).

## 4. Discussion

*C. urealyticum* is a well-known opportunistic uropathogen that in 60% of cases can occur with cystitis, pyelonephritis, and urinary sepsis [27,30]. According to a study published in 2015 by Sánchez-Martín et al., cases of *C. urealyticum* infection would have increased by 300% from 2009 to 2014, of which about 67% manifested as pyelitis [31].

Although a wide variety of coryneform bacteria have been found from the male genital tract of prostatitis patients [32,33,34,35,36], only one clinical case of prostatitis caused by *C. urealyticum* has been reported [8]. These data seemed underestimated, and led to questioning as to whether *C. urealyticum* could be one of the causative agents of chronic prostatitis. Additionally, the question arose as to whether the lack of epidemiological data about *C. urealyticum* and prostatitis is due to its peculiar characteristics of in vitro cultivation.

Chronic idiopathic bacterial prostatitis presents with less evident symptoms compared to acute bacterial prostatitis that occurs with the signs of a classic infection, and its etiology can be attributed to a bacterial origin [37].

The proposed pathophysiology involves the infiltration of pathogenic urobacteria capable of overcoming the immune defences of the prostate gland through specific virulence factors [38].

A known bacterial infection route concerns the association between bacterial prostatitis and urinary tract infection, and infection of the prostate gland can occur following an ascending urethral infection or by the reflux of infected urine into the prostate ducts [37].

Common isolates of bacterial prostatitis include the uropathogenic species *Proteus* and *Klebsiella*; like *C. urealyticum*, these bacteria have urease activity, however, they do not cause encrustation [39]. Nonetheless, urease activity is considered an important virulence factor involved in UTI and bacterial prostatitis pathogenesis [40].

These interesting analogies agree with our experimental results about *C. urealyticum* in vitro adhesion on the LNCaP prostate cell line. For the first time, we have demonstrated *C. urealyticum* tropism for prostate tissue and the involvement of the AHA as a urease inhibitor in the adhesion process. Although more in-depth studies to clarify the mechanism of action of AHA and the role of the urease enzyme are needed, our in vitro assays have shown that AHA affects numerous pathogenicity mechanisms of *C. urealyticum* in a concentration-dependent manner.

The effect of the AHA urease inhibitor on the different stages (lag phase, exponential growth, and stationary phase) of in vitro growth of the three strains of *C. urealyticum* was made by adapting a mathematical model (sigmoidal dose-response with variable slope function) to the curve data obtained by measuring the optical density (OD) of bacteria population [28,41].

Table 1 illustrates the effects of AHA on the in vitro bacterial grown curve analysis for three strains of *C. urealyticum* tested. Although all three tested strains showed a dose-dependent trend, both the percentage inhibition and the delay in reaching the exponential phase were more evident for the ATCC 43043 strain (from 13.80% to 16.26% for the percentage inhibition and from 5.16 to 11.38 hours to reach the exponential phase). On the other hand, the ATCC 43044 strain was more insensitive to treatment with AHA for both concentrations tested (from 0.52% to 1.04% for the percentage inhibition and from 0.80 to 1.09 for the delay in reaching the exponential phase). With regard to the ATCC 43042 strain, it showed intermediate values of both percentage inhibition and delay in reaching the exponential phase with respect to the other two strains (from 4.09% to 2.45% for inhibition and from 3.63 to 2.30 hours to reach the exponential phase). The different growth behavior of the three strains when tested with the two concentration of AHA could be related to differences in their metabolism. According to Schultz et al., the lag phase can be interpreted as an adaptation period in which the bacterial cell must pass from low to high metabolism. Urease serves as a nitrogen source provider for bacterial growth, in this regard the genus *Corynebacteria* is well known for the nitrogen metabolism and control [42]. *C. urealyticum* grows under the stimulation of the urea present in the urine when it adheres to the urinary tract [4]; therefore, the irreversible block of urease enzyme by AHA may influence the bacteria metabolism in a strain-dependent manner. In addition, a work by Nerving et al. in 1976 that assessed the effect of several AHAs on the growth of *Corynebacterium renale*, a pathogenic bacterium that causes cystitis and pyelonephritis, demonstrated an extension of the in vitro lag phase related to the chemical structure of the AHA and the dose tested [43].

Comparing the results obtained by analyzing the in vitro growth curves with the other assays carried out (Table 2), it is interesting to highlight that inhibitor, at both concentrations tested, did not influence the mitochondrial respiration of the two cell lines LNCaP and HeLa, either alone (uninfected) or when infected with the three strains of *C. urealyticum* In particular, LNCaP cells infected with the ATCC 43044 stain, which had shown greater indifference to AHA in the in vitro growth curves assay, showed a slight increase in mitochondrial respiration rate following the treatment with AHA at both tested concentrations.

Indeed, MTT assay relied on the ability of mitochondrial reductase to convert the tetrazolium to formazan. This reaction is related to the number of viable cells. The slight enhancement of mitochondrial respiration rate, observed on LNCaP cells simultaneously infected with *C. urealyticum* 43044 and treated with 0.25 and 0.50 mg/mL of AHA compared to untreated and uninfected cells could have been due to the ability of AHA to inhibit bacterial urease activity. Because this is an enzyme causing cell damage, its inhibition results in a cell viability increment [44].

The same strain showed a higher capacity to produce biofilm and adhesion values compared to the other tested strains. ATCC 44043 strain was a lower biofilm former (although after AHA treatment the value remained >4 and therefore strongly adherent) and the least adherent to the LNCaP cell line. Additionally, in this case, a possible explanation of the AHA strain-dependent effect on the in vitro biofilm formation can be explained by its ability to modulate the metabolism. Indeed, there are multiple pieces of evidence that relate nitrogen metabolism to biofilm formation [45]. From all these shreds of evidence, it can be deduced that the pathogenic mechanisms of *C. urealyticum* are highly strain-dependent and need further in-depth studies. Nevertheless, these preliminary in vitro tests on three strains of *C. urealyticum* represent a first starting point to understand their involvement in chronic idiopathic prostatitis.

The importance of the correct identification of a pathogen during infection does not succeed only in the epidemiological data, but above all in the more appropriate prescription of the antibiotic therapy.

Unfortunately, in the case of encrusted cystitis as well as prostatitis, antibiotic therapy alone is ineffective; indeed, in order to properly eradicate the bacterium, in addition to specific antibiotic treatment, it is essential to eliminate calcifications [8]. In this context, our study has brought new pieces of evidence on the therapeutical use of the irreversible urease inhibitors such as AHA, not only as enzyme blockers to facilitate the removal of encrustations, but also as modulators of some pathogenic mechanisms.

## 5. Conclusions

This study provided interesting preliminary data that allow us to assert that there is a real possibility that *C. urealyticum* is a new candidate for chronic idiopathic prostatitis. Furthermore, the lack of epidemiological data to support this hypothesis is to be found in the failure to identify this bacterium in routine microbiological examinations, mainly due to its particular in vitro culture needs.

## Figures and Tables

**Table 1:** In vitro bacterial grown curve analysis.

	*Corynebacterium urealyticum* ATCC 43042			*Corynebacterium urealyticum* ATCC 43043			*Corynebacterium urealyticum* ATCC 43044		
	^a^ 0.50	^a^ 0.25	^b^ C+	^a^ 0.50	^a^ 0.25	^b^ C+	^a^ 0.50	^a^ 0.25	^b^ C+
Goodness of Fit *R*^2^	0.9990	0.9997	0.9983	0.9992	0.9992	0.9994	0.9974	0.9973	0.9984
Y_top_ (CFU/mL) at 72 h	9.38 × 10^8^	9.54 × 10^8^	9.78 × 10^8^	8.19 × 10^8^	8.43 × 10^8^	9.78 × 10^8^	9.48 × 10^8^	9.53 × 10^8^	9.58 × 10^8^
Y_top_ ± SD ^c^ (CFU/mL) at 72 h	1.01 × 10^6^	1.74 × 10^6^	4.56 × 10^6^	6.39 × 10^6^	3.93 × 10^6^	3.58 × 10^6^	2.91 × 10^6^	2.90 × 10^6^	2.23 × 10^6^
Inhibition (%)	**4.09**	**2.45**	/	**16.26**	**13.80**	/	**1.04**	**0.52**	/
X_CFU50_ (h)	41.85	40.52	38.22	58.50	52.28	47.12	20.31	20.02	19.22
X_CFU50_ ± S.D. ^c^ (h)	0.02	0.05	0.15	0.12	0.09	0.09	0.01	0.01	0.07
∆X_CFU50_ (h)	**3.63**	**2.30**	/	**11.38**	**5.16**	/	**1.09**	**0.80**	/

^a^ Acetohydroxamic acid (AHA) concentration (mg/mL). ^b^ Untreated control. ^c^ Standard deviation.

**Table 2:** Results of the effect of AHA on in vitro tests for *C. urealyticum* strains tested.

*C. urealyticum strain*	Growth			Biofilm Formation			LNCaP Adhesion			HeLa Adhesion		
	^a^ C+	AHA		^a^ C+	AHA		^a^ C+	AHA		^a^ C+	AHA	
		^b^ 0.25	^b^ 0.50		^b^ 0.25	^b^ 0.50		^b^ 0.50	^b^ 0.25		^b^ 0.25	^b^ 0.50
ATCC 43042	++	++	++	++	++	++	+++	++	++	+	++	++
ATCC 43043	+	+++	+++	++	++	++	+	++	++	+	++	++
ATCC 43044	+++	+	+	+++	++	++	+++	++	++	+	++	++

^a^ Control strains not treated with AHA. ^b^ Concentrations in mg/mL. + mild effect. ++ moderate effect. +++ strong effect.

## References

[1] Bernard K.A., Funke G. (2015). Corynebacterium. Bergey’s Manual of Systematics of Archaea and Bacteria.

[2] González-Enguita C., Vela-Navarrete R. (2017). Corynebacterium urealyticum: The historical importance of its discovery. Actas Urol. Esp..

[3] Bernard K. (2012). The genus Corynebacterium and other medically relevant coryneform-like bacteria. J. Clin. Microbiol..

[4] Salem N., Salem L., Saber S., Ismail G., Bluth M.H. (2015). Corynebacterium urealyticum: A comprehensive review of an understated organism. Infect. Drug Resist..

[5] Costales J., Alsyouf M., Napolitan P., Wang S., Hu B. (2019). Corynebacterium urealyticum: Rare urinary tract infection with serious complications. Can. J. Urol..

[6] François J. (1914). La cystite incrustée. J. Urol. Med. Chair.

[7] El Sayegh H., Elouardani M., Iken A., Nouini Y., Lachkar A., Benslimane L., Belahnech Z., Faik M. (2008). Cystite incrustante à Corynebacterium urealyticum. La Revue de Médecine Interne.

[8] Pardo M.A., Rosino S.A., Rivero G.Á., Barceló B.I. (2018). Encrusted prostatitis by Corynebacterium urealyticum: The importance of clinical suspicion. Actas Urol. Esp..

[9] López-Medrano F., García-Bravo M., Morales J., András A., San Juan R., Lizasoain M., Aguado J. (2008). Urinary tract infection due to Corynebacterium urealyticum in kidney transplant recipients: An underdiagnosed etiology for obstructive uropathy and graft dysfunction—results of a prospective cohort study. Clin. Infect. Dis..

[10] Riegel P. (2006). Actualités de l′ épidémiologie et du rôle pathogène des corynébactéries. Antibiotiques.

[11] Soriano F., Tauch A. (2008). Microbiological and clinical features of Corynebacterium urealyticum: Urinary tract stones and genomics as the Rosetta Stone. Clin. Microbiol. Infect..

[12] Gómez-Garcés J.-L., Alos J.-I., Tamayo J. (2007). In vitro activity of linezolid and 12 other antimicrobials against coryneform bacteria. Int. J. Antimicrob. Agents.

[13] Soriano F., Huelves L., Naves P., Rodríguez-Cerrato V., del Prado G., Ruiz V., Ponte C. (2009). In vitro activity of ciprofloxacin, moxifloxacin, vancomycin and erythromycin against planktonic and biofilm forms of Corynebacterium urealyticum. J. Antimicrob. Chemother..

[14] Ramos J.N., Valadão T.B., Baio P.V.P., Mattos-Guaraldi A.L., Vieira V.V. (2019). Novel mutations in the QRDR region gyrA gene in multidrug-resistance Corynebacterium spp. isolates from intravenous sites. Antonie Van Leeuwenhoek.

[15] Souza C.D., Faria Y.V., Sant’Anna L.D.O., Viana V.G., Seabra S.H., Souza M.C.D., Vieira V.V., Hirata Júnior R., Moreira L.D.O., Mattos-Guaraldi A.L.D. (2015). Biofilm production by multiresistant Corynebacterium striatumassociated with nosocomial outbreak. Memorias do Instituto Oswaldo Cruz.

[16] Charalabopoulos K., Karachalios G., Baltogiannis D., Charalabopoulos A., Giannakopoulos X., Sofikitis N. (2003). Penetration of antimicrobial agents into the prostate. Chemotherapy.

[17] Elyasi S., Khalili H., Dashti-Khavidaki S., Mohammadpour A. (2012). Vancomycin-induced nephrotoxicity: Mechanism, incidence, risk factors and special populations. A literature review. Eur. J. Clin. Pharmacol..

[18] Wang H., Chen Z., Zhu Y., Wang T., Wu X. (2006). Penetrability and therapeutic effect of vancomycin to the prostates of rats with bacterial prostatitis (BP) or BPH-BP. Zhonghua Nan Ke Xue Natl. J. Androl..

[19] Marty N., Agueda L., Lapchine L., Clave D., Henry-Ferry S., Chabanon G. (1991). Adherence and hemagglutination ofCorynebacterium group D2. Eur. J. Clin. Microbiol. Infect. Dis..

[20] Mandlik A., Swierczynski A., Das A., Ton-That H. (2007). Corynebacterium diphtheriae employs specific minor pilins to target human pharyngeal epithelial cells. Mol. Microbiol..

[21] Tauch A., Trost E., Tilker A., Ludewig U., Schneiker S., Goesmann A., Arnold W., Bekel T., Brinkrolf K., Brune I. (2008). The lifestyle of Corynebacterium urealyticum derived from its complete genome sequence established by pyrosequencing. J. Biotechnol..

[22] Richmond S., Yep A. (2019). Quantification of Urease Activity. Proteus Mirabilis.

[23] Genovese C., Davinelli S., Mangano K., Tempera G., Nicolosi D., Corsello S., Vergalito F., Tartaglia E., Scapagnini G., Di Marco R. (2018). Effects of a new combination of plant extracts plus d-mannose for the management of uncomplicated recurrent urinary tract infections. J. Chemother..

[24] Sabiote L., Emiliani E., Kanashiro A.K., Balañà J., Mosquera L., Sánchez-Martín F.M., Millán F., Alonso C., Palou J., Angerri O. (2020). Oral Acidification with l-Methionine as a Noninvasive Treatment for Encrusted Uropathy. J. Endourol. Case Rep..

[25] Miano R., Germani S., Vespasiani G. (2007). Stones and urinary tract infections. Urol. Int..

[26] Rahman N.U., Meng M.V., Stoller M.L. (2003). Infections and urinary stone disease. Curr. Pharm. Des..

[27] Cappuccino L., Bottino P., Torricella A., Pontremoli R. (2014). Nephrolithiasis by Corynebacterium urealyticum infection: Literature review and case report. J. Nephrol..

[28] Fuochi V., Li Volti G., Camiolo G., Tiralongo F., Giallongo C., Distefano A., Petronio Petronio G., Barbagallo I., Viola M., Furneri P.M. (2017). Antimicrobial and anti-proliferative effects of skin mucus derived from Dasyatis pastinaca (Linnaeus, 1758). Mar. Drugs.

[29] Gomes D.L., Martins C.A., Faria L.M., Santos L.S., Santos C.S., Sabbadini P.S., Souza M.C., Alves G.B., Rosa A.C., Nagao P.E. (2009). Corynebacterium diphtheriae as an emerging pathogen in nephrostomy catheter-related infection: Evaluation of traits associated with bacterial virulence. J. Med. Microbiol..

[30] Lieten S., Schelfaut D., Wissing K.M., Geers C., Tielemans C. (2011). Alkaline-encrusted pyelitis and cystitis: An easily missed and life-threatening urinary infection. Case Rep..

[31] Sánchez-Martín F., López-Martínez J., Kanashiro-Azabache A., Moncada E., Angerri-Feu O., Millán-Rodríguez F., Villavicencio-Mavrich H. (2016). Corinebacterium urealyticum: Increased incidence of infection and encrusted uropathy. Actas Urológicas Españolas.

[32] Netzel T. (2002). Prostatitis associated with Corynebacterium glucuronolyticum. Clin. Microbiol. Newslett..

[33] Magri V., Trinchieri A., Ceriani I., Marras E., Perletti G. (2007). Eradication of unusual pathogens by combination pharmacological therapy is paralleled by improvement of signs and symptoms of chronic prostatitis syndrome. Arch. Ital. Urol. Androl..

[34] Ivanov I.B., Kuzmin M.D., Gritsenko V.A. (2009). Microflora of the seminal fluid of healthy men and men suffering from chronic prostatitis syndrome. Int. J. Androl..

[35] Türk S., Punab M., Mändar R. (2009). Antimicrobial susceptibility patterns of coryneform bacteria isolated from semen. Open Infect. Dis. J..

[36] Türk S., Mazzoli S., Štšepetova J., Kuznetsova J., Mändar R. (2014). Coryneform bacteria in human semen: Inter-assay variability in species composition detection and biofilm production ability. Microb. Ecol. Health Dis..

[37] Lummus W.E., Thompson I. (2001). Prostatitis. Emerg. Med. Clin. N. Am..

[38] Khan F.U., Ihsan A.U., Khan H.U., Jana R., Wazir J., Khongorzul P., Waqar M., Zhou X. (2017). Comprehensive overview of prostatitis. Biomed. Pharmacother..

[39] Davis N.G., Silberman M. (2019). Bacterial Acute Prostatitis.

[40] Delcaru C., Alexandru I., Podgoreanu P., Grosu M., Stavropoulos E., Chifiriuc M.C., Lazar V. (2016). Microbial biofilms in urinary tract infections and prostatitis: Etiology, pathogenicity, and combating strategies. Pathogens.

[41] Ram Y., Dellus-Gur E., Bibi M., Karkare K., Obolski U., Feldman M.W., Cooper T.F., Berman J., Hadany L. (2019). Predicting microbial growth in a mixed culture from growth curve data. Proc. Natl. Acad. Sci. USA.

[42] Walter B., Hänssler E., Kalinowski J., Burkovski A. (2007). Nitrogen metabolism and nitrogen control in corynebacteria: Variations of a common theme. J. Mol. Microbiol. Biotechnol..

[43] Nervig R.M., Kadis S. (1976). Effect of hydroxamic acids on growth and urease activity in Corynebacterium renale. Can. J. Microbiol..

[44] Kappaun K., Piovesan A.R., Carlini C.R., Ligabue-Braun R. (2018). Ureases: Historical aspects, catalytic, and non-catalytic properties—A review. J. Adv. Res..

[45] Rinaldo S., Giardina G., Mantoni F., Paone A., Cutruzzolà F. (2018). Beyond nitrogen metabolism: Nitric oxide, cyclic-di-GMP and bacterial biofilms. FEMS Microbiol. Lett..

